# Suspended Sediments Quality Assessment in a Coastal River: Identification of Potentially Toxic Elements

**DOI:** 10.3390/ijerph19074293

**Published:** 2022-04-03

**Authors:** Jie Zeng, Guilin Han, Shitong Zhang, Qian Zhang

**Affiliations:** 1 Institute of Earth Sciences, China University of Geosciences (Beijing), Beijing 100083, China; zengjie@cugb.edu.cn (J.Z.); stongzhang0103@cugb.edu.cn (S.Z.); 2 Institute of Geographic Sciences and Natural Resources Research, Chinese Academy of Sciences, Beijing 100101, China; zhangqian@igsnrr.ac.cn

**Keywords:** suspended particulate matter (SPM), potentially toxic elements (PTE), pollution source and evaluation, Jiulongjiang River

## Abstract

In coastal rivers with various human and damming activities (reservoir), the cycle and biogeochemistry of environmental pollutants in river systems has been modified. A total of 42 suspended particulate matter (SPM) samples were obtained in Jiulongjiang River, southeast China to investigate the concentration, sources, behavior, and risks of nine potentially toxic elements (PTEs) in SPM. The results of metals concentration showed relatively large variation, major for Mn and minor for Co; Mn > Zn > V > Pb > Cr > Ni > Cu > Cd > Co. Multi-index evaluation reflected that most of the PTEs are minor enrichment/moderately polluted. The Cd is defined as extremely severe enrichment/polluted level, and the Pb and Zn as minor enrichment/moderately polluted levels. Among the selected PTEs, Cd and Zn are identified as the main toxic factors of SPM with a contribution of 57 ± 18% and 14 ± 7% to the total toxic risk. The sources identification suggested that human inputs may be the primary potential source of Cd, Zn, Pb, and Co, whereas natural sources (e.g., rock weathering) are likely to be responsible for Cu, Cr, V, and Ni. In contrast, the data suggested that Mn may be attributed to both natural and anthropogenic inputs. The PTEs among dissolved, suspended, and sediment phases reflected the transportation behavior and different potential risk levels. Overall, the PTE geochemistry of river SPM can act as a good indicator of the driving mechanism of PTEs’ accumulation and provide a powerful support for controlling riverine PTEs-related pollution in coastal regions.

## 1. Introduction

It is vital to evaluate the contamination level of rivers given the background of the worsening status of the global hydrosphere, and this would be of benefit for the management of water resources [1,2,3]. Coastal rivers are the most important pathways linking the ocean and land, which influences various biogeochemical processes. There are numerous issues occurring in coastal rivers due to water resources overuse and industrial/agricultural waste discharge [4]. The anthropogenic pollutants impacting coastal river sediments (suspended and bed sediments) are of growing concern [5]. Potentially toxic elements (PTEs) are one of the representative contaminants [6]. The PTEs tend to possess features of non-biodegradability, bio-accumulation, and acute/chronic toxicity [7,8,9,10], are harmful to aquatic eco-environment and even pose a threat to human health [11,12]. Both the anthropogenic input (e.g., industrial/domestic wastes) and natural process (e.g., soil erosion and weathering process) are the sources of PTEs in the coastal river water environment [13,14,15]. Moreover, PTEs exist in rivers in three phases: dissolved loads, bed sedimentary loads, and suspended loads [16].

Suspended particulate matter (SPM or suspended sediment) is an important adsorption carrier for PTEs in coastal river systems, and can efficiently absorb dissolved PTEs due to its high surface activity [14,17]. There are two origins of fluvial SPM: rainfall-driven soil erosion products and bed sediment re-suspended matter [18,19]. In coastal river systems, the SPM is the most important contributor (about 90%) of the export of terrestrial matters into the sea [20]. Riverine SPM is also a good indicator of basin pollution, indicating the natural inputs and anthropogenic inputs, and reflecting further the potential risk or negative effect on life. Moreover, the SPM-adsorbed PTEs may be re-released into river water again (secondary pollution) if environmental settings change (e.g., salinity, redox potential, pH). Therefore, exploring and clarifying particulate PTEs’ pollution and its source are significant, and many studies have reported particulate PTEs all over the world [18,21,22], contributing to the safety assessment and high-efficiency management of river water environments.

As one of the most important coastal rivers in southern Fujian Province (China), Jiulongjiang River basin contributes to the development of various land ecosystems and supports water resources for millions of inhabitant [23,24]. Moreover, Jiulongjiang River is an ideal region for clean hydroelectric production because of the abundant flow and steeply sloping river [24,25], which can strongly support the local economy. The development of hydropower dams, together with other anthropogenic activities (e.g., urban emission and agricultural production), have significantly changed hydrodynamic conditions (e.g., hydraulic residence time, HRT) and further impacted the eco-environmental processes and biogeochemical cycling of the coastal river water [26,27]. In addition, the concentration of PTEs in dissolved phases and sediments has also exhibited obvious spatial variations from upstream to downstream of typical cascade dams [28]. Moreover, the PTE concentrations vary in the river system due to changes in some environmental factors, such as water temperature, pH, redox potential, and rainfall. These environmental factors can realize PTEs; redistribution in the river system by influencing adsorption, precipitation and resuspension processes [12,18].

To date, PTE-related studies of the coastal Jiulongjiang River basin (JRB) have mainly focused on dissolved and sediment phases in estuarial areas and the mainstream [24,29]. These studies reported that the dissolved PTEs in JRB can be defined as dominant (>100 μg L^−^^1^), moderately abundant (10~100 μg L^−^^1^), and less abundant (<10 μg L^−^^1^) elements according to their concentrations. The major sources of dissolved PTEs were agricultural inputs, natural weathering and urban human activities [29]. In contrast, the distribution pattern of PTEs’ contents in river sediment highly matched the reservoir/dam distribution along the JRB. The dominant ecological risks of Cd and Pb in sediments were also observed [24]. However, the status, pollution, risks, and sources of PTEs in suspended sediments (SPM) and their relationship with dissolved PTEs and PTEs in sediments have been rarely reported in the dams distributed along coastal rivers.

This study performed a field sampling in the coastal Jiulongjiang River during July 2017 to improve knowledge of the river’s environmental particulate PETs. Forty-two SPM samples were collected and measured for PETs. The major purposes are to: (1) distinguish the particulate PTEs’ concentration, pollution, and related risk, (2) explore the PTEs’ origins, and (3) clarify the particulate PTEs’ behavior among the dissolved, suspended sediments, and the bed sediment. This work supports a data base for the management of coastal river basins and provides significant reference for similar river systems.

## 2. Materials and Methods

### 2.1. Regional Background

**Figure 1:**
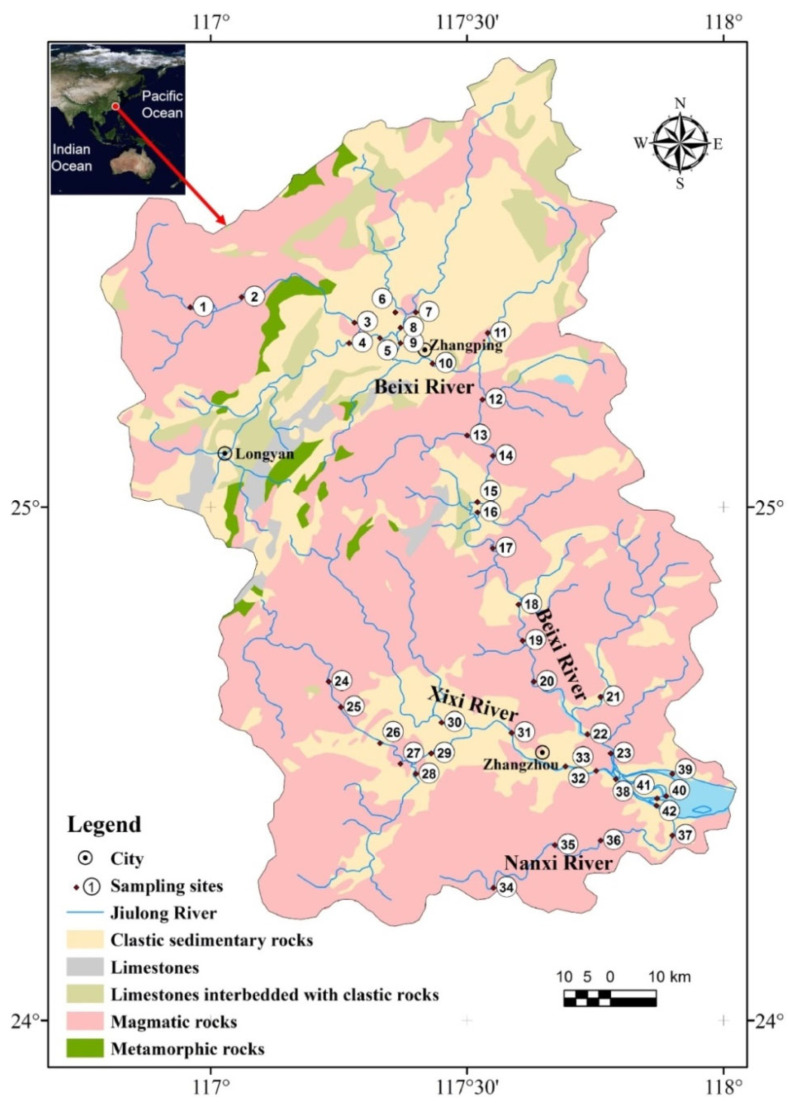
Sample sites’ distribution and lithology for JRB.2.2. Sampling and Chemical Analysis.

The Jiulongjiang River is the largest coastal river in the territory of southern Fujian Province, Southeast China (Figure 1). The mainstream originates from the northwest (Longyan city) and runs southeast (Zhangzhou city). Then, the Jiulongjiang River flows into the Xiamen Bay [30]. The Jiulongjiang River basin (JRB) presents a wide area (~1.47 × 10^4^ km^2^) with a mainstream (the Beixi River) and two major tributaries (the Xixi River and the Nanxi River) [29]. The tributaries are relatively short in length. The JRB contains diverse geography and landscapes, and shows a varied altitude (<50~1800 m). Mountains, hills, and plains are found in the LRB. Regarding land use, the JRB mainly has forest land (~63.5%), grass land (~15.7%), cropland (~14.4%), an urban area (~5.4%), water area (~0.9%), and unused land (~0.1%). Red soils are the major soil type within the basin (>90% basin area). The JRB is influenced by a sub-tropical monsoon climate with an annual temperature of 20~21 °C in the whole basin. The annual rainfall varies from 1400 to 1800 mm [31]. The lithology of the JRB is mainly composed of clastic sedimentary and magmatic rocks, with rarely distributed limestones and metamorphic rocks. Mining areas (e.g., Fe mine) are mainly distributed in the northwest basin [32]. The upper reaches are mainly covered by forest and mountain areas, while the lower reaches are affected by different human activities, such as agriculture and industry. Moreover, several reservoirs/dams are distributed within the JRB, such as the Wananxi Reservoir, which significantly regulates the fluvial discharge [24].

In the field work, according to the population distribution, land cover, and diverse lithology, 42 sites in the JRB were chosen to conduct sampling in July 2017. Although the samples were only collected in wet season, considering that the object of this study is suspended sediments (SPM) and the river suspended sediments’ transport generally occurs in the wet season (~90%), the contribution of the dry season is relative negligible. Therefore, the sampling is representative for the SPM study. Among the sampling sites, site 1~21, 24~33, and 34~37 were distributed in the Beixi River, the Xixi River, and the Nanxi River, respectively. Site 22, 23, and 38~42 were located in the estuary area (Figure 1). In total, 42 riverine water samples were obtained, and the samples were further filtered by the 0.22 μm cellulose acetate membranes to separate SPM samples within 24 h. After field work, the SPM samples were further digested. The detailed digestion process is described in our previous work [31,33]. In brief, the digestion of SPM sample powder (weighted) was conducted in a pre-clean jar via mixed HF/HNO_3_ (*v/v* = 1/3) acid under 140 °C. Next, the digested solution was dried and the removal of fluoride was performed by HNO_3_ (2 mL, twice). Furthermore, the digested solution was quantitatively dissolved in 2% HNO_3_ for final analysis. Double-distilled acid (ultra-pure acid) was applied in the whole digestion process [8]. Nine PTEs of all samples were detected via ICP-MS (Elan DRC-e, PerkinElmer, Waltham, MA, USA). The procedural blank, repeated samples, and standard reference material (SRM, GBW 07447) were used during the measuring procedure. The relative standard deviation of replicate detection was ±5%, and the PTEs’ recoveries of SRM > 95%.

### 2.2. Appraisal Methods

#### 2.2.1. PTEs’ Pollution Assessment

The PTEs’ concentration of SPM samples are normalized to the environmental conservative element (Al) and further compared with the natural background to assess the PTE enrichment level, i.e., the Enrichment factor (EF) calculation [34,35]. The detailed calculation of EF is as follows. The related classification of the enrichment level is listed in Supplementary Materials (Table S1). The data of Al concentrations are derived from our previous work [31].

(1) $$\mathrm{EF}=\frac{{{(C}_{i}{/C}_{\mathrm{ref}})}_{\mathrm{SPM}}}{{{(C}_{i}{/C}_{\mathrm{ref}})}_{\mathrm{background}}}$$

where C_i_ is the PTEs concentration (mg kg^−1^), and C_ref_ is the concentration of the reference element (Al, mg kg^−1^). The (C_i_/C_ref_)_background_ is the ratio of background content values of PTEs and reference element obtained from the upper continental crust (UCC, with the contents of Al = 81,506 mg kg^−1^, V = 97 mg kg^−1^, Cr = 92 mg kg^−1^, Mn = 774.5 mg kg^−1^, Co = 17.3 mg kg^−1^, Ni = 47 mg kg^−1^, Cu = 28 mg kg^−1^, Zn = 67 mg kg^−1^, Cd = 0.09 mg kg^−1^, Pb = 17 mg kg^−1^).

Furthermore, the particulate PTEs contamination level is assessed via the geo-accumulation index (I_geo_) as follows [36]:

(2) $$I_{\mathrm{geo}}{=\log}_{2}(\frac{C_{i}}{1{.5\times U}_{i}})$$

where C_i_ is the PTEs content of SPM (mg kg^−1^), U_i_ is the corresponding PTEs content of the UCC (mg kg^−1^), and 1.5 is the correction factor. Accordingly, the I_geo_ can also be classified as 7 classes of pollution level (Table S1).

The pollution load index (PLI) based on contamination factor (CF) is also applied to quantitatively evaluate the total pollution levels of PTEs [37], as follows:

CF = C_s_/C_b_ (3)

PLI = (CF_1_×CF_2_×CF_3_×…×CF_n_)^1/n^ (4)

Here, C_s_/C_b_ is the ratio of river particulate PTEs content and the upper continental crust PTEs content. If PLI less than 1, the combined contamination is negligible, whereas particulate PTEs’ contamination exists when PLI > 1 [38].

#### 2.2.2. PTEs‘ Risk Assessment 

The toxic risk index (TRI) is chosen to assess total toxic risk of particulate PTEs to potential exposed aquatic organisms and/or humans. This index considered both the threshold and the probable effect level (TEL and PEL), confirmed by previous studies [39,40]. The literature-certified TEL and PEL values are adopted to ensure a reliable risk for the assessment results [41]. The TRI classifications are listed in Table S1 and the calculation process of TRI is as follows [39]:

(5) $$\mathrm{TRI}=\overset{n}{\underset{i=1}{\sum }}{\mathrm{TRI}}_{i}=\sqrt{\frac{{\left(S_{s}^{i}/S_{\mathrm{TEL}}^{i}\right)}^{2}+{\left(S_{s}^{i}/S_{\mathrm{PEL}}^{i}\right)}^{2}}{2}}$$

where $S_{s}^{i}$ represent PTEs’ content of SPM (mg kg^−1^), $S_{\mathrm{TEL}}^{i}$ is the TEL (threshold effect level) value and $S_{\mathrm{PEL}}^{i}$ is the PEL (probable effect level) value of each PTE (mg kg^−1^).

#### 2.2.3. Particulate PTEs’ Transported Percentage

According to previous studies, the migration behavior of riverine PTEs can be indicated by the fraction (f, in %) calculation of particulate PTEs’ transported percentage [8], as follows:

(6) $$f=\frac{{\mathrm{SPM}\;\times \;C}_{p}}{{\mathrm{SPM}\;\times \;C}_{p\;}{+C}_{d}}\;\times \;100$$

where the C_p_, C_d_, and SPM are the median values of particulate PTE concentration, dissolved PTE concentration, and SPM concentration, respectively. The data of SPM and dissolved PTE concentration were derived from our previous work [31,42]. 

## 3. Results and Discussion

### 3.1. PTEs’ Concentrations of Fluvial SPM

Nine PTEs’ concentrations of SPM in the whole Jiulongjiang River, Beixi River, Xixi River, Nanxi River, and the estuary are summarized in Table 1. All concentrations (in mg kg^−1^) varied within a large range including V: 47.0~179.2, Cr: 14.9~271.4, Mn: 577.6~3041.7, Co: 4.5~23.5, Ni: 9.1~91.0, Cu: 6.0~98.5, Zn: 77.0~1111.2, Cd: 7.5~30.8, Pb: 10.3~125.0. In the whole basin, the median PTEs’ concentrations are appropriate for comparison. As listed in Table 1, the studied particulate PTEs’ concentration presented in the order of Mn > Zn > V > Pb > Cr > Ni > Cu > Cd > Co, which is different from the sequences observed in other Asian and global rivers [14,22,43]. The variations in these sequences could be attributed to diverse lithological settings and the particulate PTEs’ abundances [38]. Obviously, the concentrations of the four PTEs (Mn, Zn, V, and Pb) with highest abundance are several to dozens of times higher than those of other PTEs. In addition to V, Cr, Co, Ni, and Cu, the contents of half of the PTEs in SPM in Jiulongjiang River exceed the UCC contents [44]. In particular, the particulate Cd and Zn concentrations are 125 and 5 times those of upper continental crust (UCC) contents, respectively.

The Jiulongjiang River showed different PTEs’ concentrations in riverine SPM because of various economic levels and industrial structures within the entire basin. From a global perspective (Table 2), the contents of V, Cr, Co, Ni, and Cu of SPM in Jiulongjiang River basin present a poor level, while the Mn concentration is similar to that of Asian (China) rivers [14,22,43]. In contrast, the typical anthropogenic emission-related PTEs, for example Cd and Zn, which are the important factors leading to river pollution, are higher than that in global rivers (average values) [14]. Among them, particulate Zn and Pb concentrations in the Jiulongjiang River basin are much closer to that in European rivers distributed in many developed countries [14]. The findings suggest that the JRB is influenced by different degrees of anthropogenic disturbances to a certain extent.

### 3.2. Appraisal of PTEs’ Pollution and Risk

#### 3.2.1. EF and I_geo_

**Figure 2:**
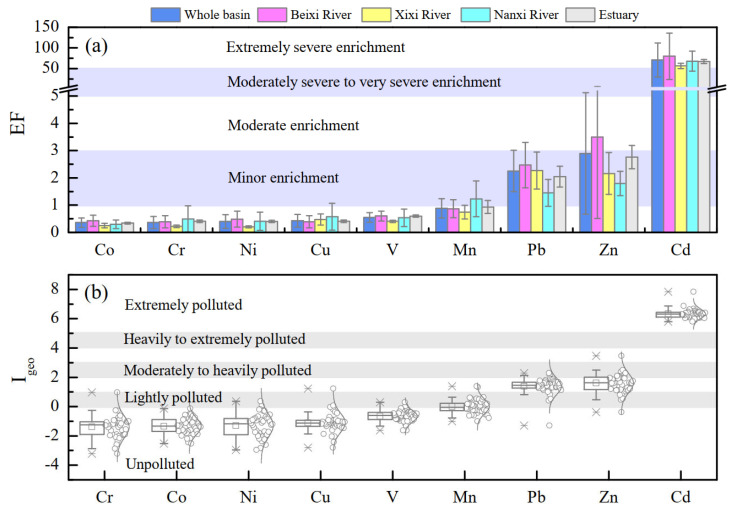
Enrichment factor (**a**) and geo-accumulation index (**b**) of particulate PTEs in JRB.

The calculated values of EF and I_geo_ of potentially toxic elements of suspended particulate matter in the Jiulongjiang River basin are plotted in Figure 2. The mean value of EF of each PTE in this study presented the order of Cd (71.1) > Zn (2.9) > Pb (2.3) > Mn (0.9) > V (0.5) > Cu (0.4) ≈ Ni (0.4) ≈ Cr (0.4) ≈ Co (0.4) in the entire basin, reflecting that Cd had an extremely severe enrichment level, followed by minor enrichment of Zn and Pb (Figure 2a and Table S1). In addition to Cd, approximately half of the samples in the JRB exhibited a relatively high EF value of Zn (>3.0) with a much higher value of up to 15.6 (site 4), which can be defined as moderate enrichment. Although Pb shows a mean EF value of 2.3, three sampling sites present the EF_Pb_ values > 3 and the highest EF_Pb_ = 5.3 (site 4), indicating moderate to moderately severe enrichment. In contrast, except for a few sites, the EF values of other PTEs (V, Cr, Mn, Co, Ni, and Cu) in most SPM samples are smaller than 1, i.e., no enrichment. Noteworthy, Cd, Zn, Pb, V, Cr, Co, and Ni consistently observed the highest EF value at site 4, and Cu and Mn also present a relatively high EF value, implying that this site is possibly affected by the strongest anthropogenic disturbances [34]. Regarding most PTEs, the mean values of EF from mainstream (Beixi River), tributaries (Xixi River, Nanxi River) and estuary are similar, while the mean values of EF_Pb_ and EF_Zn_ are highest in Beixi River and lowest in Nanxi River (Figure 2a). Large changes in EF value are observed in several PTEs (e.g., Zn, and Cd) with high standard deviation (SD = 2.2~41.1, Figure 2a), while little changes are presented in Co, Cr, and Cu. These findings suggest the spatial heterogeneity of various possible origins of particulate PTEs (human emission/natural processes) [22]. Compared with a background area with limited anthropogenic disturbance (e.g., Tibetan Plateau region, EF_Cd_ = 1.4, EF_Pb_ = 0.8, EF_Zn_ = 0.7) [38], the enrichment of particulate PTEs of the Jiulongjiang River is much serious. Most particulate PTEs present different levels of EF values in Jiulongjiang River compared to that in the agriculture-controlled basin in the tropical zone, Mun River, with EF values of Cd (17.5), Mn (14.3), Zn (5.8), Cr (1.4), Ni (1.4), Cu (1.0), Pb (0.9), V (0.9) [22], but consistently high EF_Cd_ and EF_Zn_ values were also found. In contrast, compared with the contaminated city riverine system with obviously high EF values of Pb (19.6), Ni (12.5), Cr (11.0), and Cu (10.0) [45], the enrichment level of most PTEs is relatively light in the Jiulongjiang River. These comparisons also reflect the important impact/contribution of economic and sociological level (city or village) and industry structure (industry or agriculture) on various potential PTEs’ sources and the further PTEs’ accumulation in fluvial SPM.

The pollution level of particulate PTEs is also evaluated via the I_geo_. As shown in Figure 2b, all I_geo_ values of PTEs are normal distribution in JRB via the Kolmogorov–Smirnov (K-S) test. Therefore, the average I_geo_ value of PTE is appropriate to be compared with each other [14,46]. The average I_geo_ value of almost all PTEs (except for Cr and Co) present a similar sequence to the EF values of the corresponding PTEs, namely, Cd > Zn > Pb > Mn > V > Cu > Ni > Co > Cr (b). According to the categories of geo-accumulation index (Table S1), the most contaminated PTE of SPM is Cd with the largest I_geo_ average value of 6.3, indicating an extremely polluted level. The following PTEs are Zn (I_geo_ = 1.6) and Pb (I_geo_ = 1.4) which can be defined as a moderately polluted level. The other PTEs (Mn, V, Cu, Ni, Co, and Cr) have the mean I_geo_ values < 0, revealing an unpolluted level (b). The findings in JRB are similar to rivers under the impact of the mining and smelting industry (I_geo_ = 7.0, 2.7, 1.5 for Cd, Zn, Pb) [17], i.e, Cd, Zn, and Pb are three polluted PTEs. However, the average I_geo_ values of PTE in JRB are relatively low [17], reflecting relatively lighter PTEs’ pollution of the fluvial SPM in the Jiulongjiang River than that in intensive industry-impacted basins. In contrast, the observed I_geo_ values of Pb, Zn, Cd in JRB are higher than that in an agricultural production influenced riverine basin, such as the Mun River, with heavily polluted Cd (I_geo_ = 3.7), moderately polluted Zn (I_geo_ = 1.4), and unpolluted Pb (I_geo_ < 0) [22]. Overall, the contamination level of particulate PTEs is impacted by various potential pollutant sources and buffered by various landscapes within the entire Jiulongjiang River basin.

#### 3.2.2. The Pollution Load Index (PLI)

The pollution load index (PLI) of PTEs in SPM was calculated and listed in Table 3. The PLI values varied between 1.0 and 4.2 (2.1 ± 0.6) with all PLI > 1, indicating the contamination of the particulate PTEs in this coastal riverine system. The largest observed PLI value was in site 37 in the Nanxi River (PLI = 4.2), followed by site 4 (PLI = 3.7), site 8 (PLI = 2.8), site 7 (PLI = 2.7), and site 9 (PLI = 2.7) in the Beixi River. In detail, site 37 is the last site before the Nanxi River enters the mainstream, while sites 4, 8, 7, and 9 are distributed in the Beixi River near Zhangping city (urban area). Therefore, the main reason for the high PLI values of some sites is that these sites are near the human-influenced region (urban area). It is notable that most of the sites along the Beixi River and estuary area present PLI values greater than 2.0. Overall, the calculated results of PLI suggest the non-negligible comprehensive contamination of the PTEs relative to the background region (e.g., the river in the Himalayan area, PLI > 1.0) [38].

#### 3.2.3. Assessment of Toxicity Risk (TRI)

**Figure 3:**
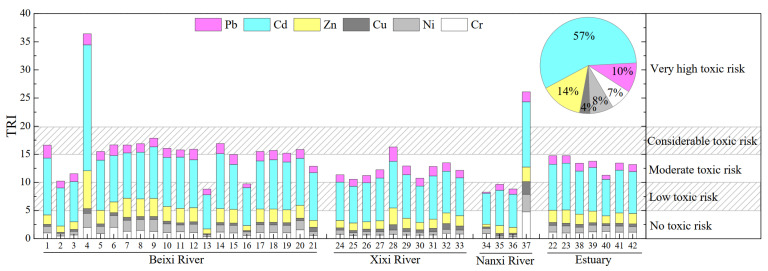
TRI values of particulate PTEs in the Jiulongjiang River.

The toxic risk index (TRI) is adopted to evaluate total particulate PTEs’ toxic risk (for fluvial aquatic organisms) [39,40], which considers the acute and chronic toxicity of related PTEs. The TRIs of all samples were calculated via available values of PEL and TEL (Table 1) as mentioned in the Methods section. As shown in Figure 3, the TRIs of six potentially toxic elements of fluvial suspended particulate matter were estimated, due to lack of a certified PEL and TEL value for the other PTEs. The TRI values range between 8.3 (site 34) and 36.4 (site 4), with a mean value of 14.3, indicating the moderate toxic risk and considerable toxic risk of the studied PTEs in most SPM samples. Moreover, sites 4 and 37 exhibited very high toxic risk, according to TRI (TRI > 20), by PTEs, in line with the assessment of pollution load index before (high PLI values, Table 3). Slightly different from the accumulation indexes (EF or I_geo_) of PTEs (Figure 2), the TRI values of each PTE are in the sequence Cd (8.2) > Zn (2.0) > Pb (1.4) > Ni (1.13) > Cr (1.06) > Cu (0.5) (Figure 3), which is caused by the different TEL/PEL values of each PTE and the SPM concentrations. Furthermore, the order of TRIs in the Jiulongjiang River is different from that of other Asia rivers, such as the Zhujiang River (large-scale basin), the Beijiang River (industry-influenced basin), and the Mun River (agriculture-controlled basin) [22,34,43]. In particular, the TRI_Cd_ values in Jiulongjiang River were much higher, indicating the heterogeneity of PTEs’ toxicity risk in the fluvial system in varied catchment landscapes and/or human activities. For the six TRI-calculated PTEs, the contributions of Cd, Zn, Pb, Ni, Cr, and Cu to the total TRI are 57 ± 18%, 14 ± 7%, 10 ± 3%, 8 ± 4%, 7 ± 5%, and 4 ± 2% (Figure 3), respectively. These results suggest Cd as the main toxic PTE of SPM in Jiulongjiang River, followed by Zn and Pb, with a contribution exceeding 10%. Therefore, given the potential species-specific toxicity of these three PTEs, for example, the nephrotoxicity and nervous system toxicity of Pb [47], and the hepatotoxicity, nephrotoxicity, and neurotoxicity of Cd [48], more attention is needed for these PTEs in the Jiulongjiang River.

### 3.3. Sources’ Identification of PTEs

**Figure 4:**
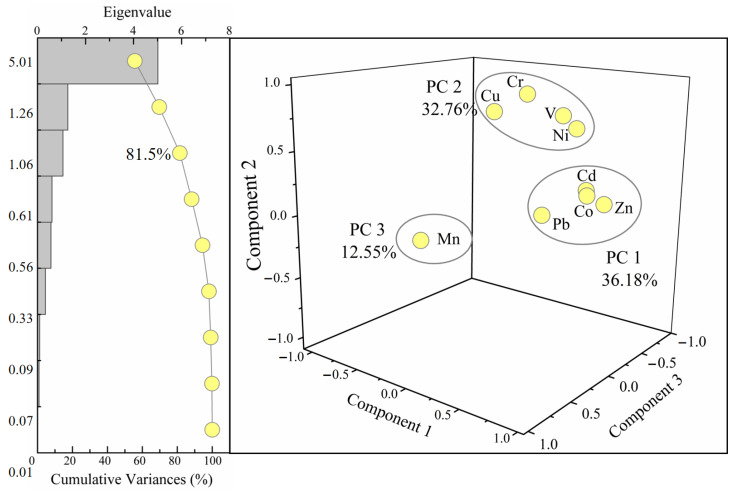
PCA results of factor loadings of PTEs of SPM in Jiulongjiang River.3.4. PTEs’ Behavior between Dissolved, Suspended and Sediment Phases.

The common multivariate statistical method principal component analysis (PCA) is employed to identify the major sources of PTEs by exploring their relationships and possible sources, decreasing the dataset dimensionality to fewer factors and preserving the associations reflected in the raw data [46]. In this study, the PCA is performed with the varimax rotation method in SPSS 21.0. The test results of Bartlett’s sphericity test and Kaiser-Meyer-Olkin test (*p* < 0.001) presented the suitability of the dataset. As shown in Figure 4, three principal components (PCs or components) are distinguished. These three PCs are extracted based on the eigenvalues exceeding 1, which account for 81.5% of the total variances. Among these, PC 1, PC 2 (Cu, Cr, V, Ni), and PC 3 (Mn) explain 36.2%, 32.8%, and 12.5% of total variances. Most of the PTEs show loading values of >0.75 within the corresponding PC, that is, strong loading.

Four PTEs (Cd, Zn, Pb, Co) exhibited component 1 with high loadings (Figure 4). Given the extremely severe enrichment of Cd (EF_Cd_ = 71.1) and relatively enriched Zn (EF_Zn_ = 2.9) and Pb (EF_Pb_ = 2.3), here we concluded that these PTEs in PC 1 are primarily attributed to the contribution of human inputs within the whole basin. As a potential explanation, the fossil fuel burning (including oil and coal) is of representative human-made Pb origin [49], the combustion behavior-originated sedimentation from the atmosphere is of important Zn origin [50], and Cd is generally observed in automobile tires and further released to the aquatic environment via wearing processes [51], which can be supported via published work [45]. Moreover, the mining activities in the upper reaches are also a potential contributor of Pb and Zn.

As shown in Figure 2 and Figure 4, the PTEs (Cu, Cr, V, Ni) in PC 2 are identified as no enrichment (EF < 1) and unpolluted (I_geo_ < 0). Although Cu, Cr, V, and Ni have the potential sources of industry wastes (mainly occurred during metal processing) and urban pollution, such as electroplate industry [52], the lower EF and I_geo_ values indicate that the human sources contributed limitary to these PTEs. Consequently, we infer that this PC is originated from natural processes which are mainly influenced by soil erosion and rock weathering processes based on the EF/I_geo_ values and positive loading of these four PTEs in this PC. 

In addition, it is noteworthy that Mn is the sole element that presented high positive loading in PC 3 (Figure 4). Given the relatively higher EF/I_geo_ values of Mn than that of Cu, Cr, V, and Ni (Figure 2), and the weak loadings of Mn in PC 1 and PC 2, PC 3 is thereby determined as a joint contribution of both natural inputs and artificial contaminant. This can also be supported through the accelerated rock/soil weathering caused by intensive urban development and construction within the river basin, which resulted in the characteristic element (Mn) export to the fluvial system in the particle phase [53].

The particulate PTEs’ transported percentages are calculated based on Equation (6). Finally, the fractions of particulate-transported V, Cr, Mn, Co, and Ni are calculated, since the dissolved concentrations of other PTEs are not available. These five PTEs present a proportion order of f_Mn_ (92.1%) > f_Cr_ (83.5%) > f_V_ (82.8%) > f_Co_ (81.6%) > f_Ni_ (54.7%) (Table 4), implying that the fluvial PTEs transportation are dominated by the SPM phase, except for Ni. Generally, the behavior of dissolved PTEs and dissolved major cations (e.g., sodium and potassium) is relatively similar, presenting a declined concentration with increasing river discharge, namely the dilution effect [54,55]. Moreover, the intensity of rainfall-driven soil erosion products’ (particulates) export to the riverine ecosystem is relatively large because of the higher rainfall amount in the rainy season [6,13]. Consequently, the fluvial PTEs in this study are more likely to be transported in particulate phase rather than the dissolved phase during the study period (rainy season). In contrast, the relative contribution of the dry season is limited due less river sediment discharge and the low PTEs’ flux.

**Figure 5:**
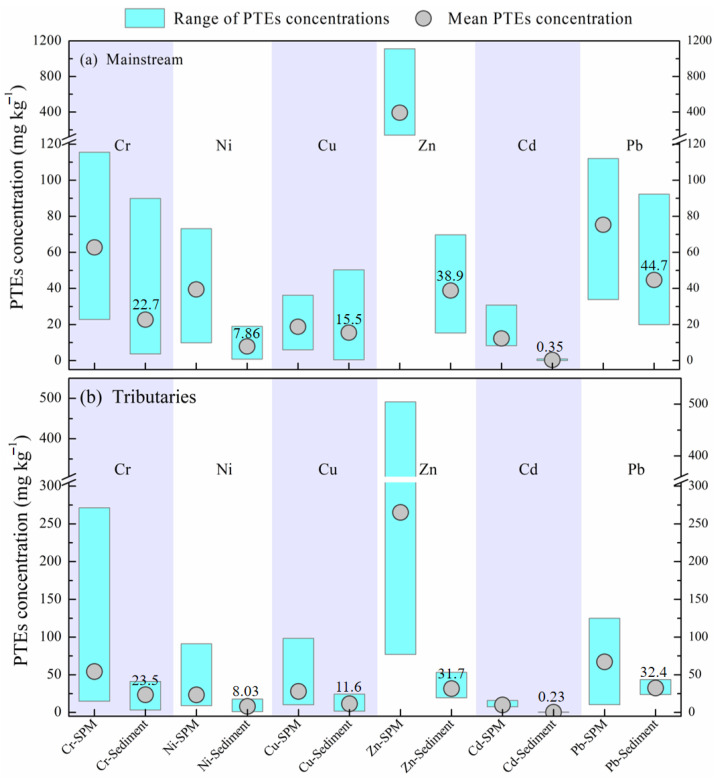
PTEs concentration between SPM and sediment in the Jiulongjiang River, (**a**) Mainstream (Beixi River), (**b**) Tributaries (Xixi and Nanxi River). Sediment data are from [24].

Although the sediment samples were not collected in this study, here we summarized and compared the recent PTEs of sediment in the Jiulongjiang River from the literature [24]. In both the mainstream and the tributaries, the concentrations of most PTEs in SPM have a wider range of variation than that in sediments (Figure 5). The mean concentration of these PTEs presented the same trend, i.e., the SPM is higher than sediment. The mean values-based PTE concentration ratios of SPM and sediments varied from 1.2 (Cu) to 35.2 (Cd) in mainstream, and from 2.1 (Pb) to 43.2 (Cd) in the tributaries. These findings indicated that the overall level of PTEs in suspended solids is higher, and the corresponding potential risks are also higher relative to sediments. As a possible explanation, SPM showed stronger adsorption capacity for exogenous input PTEs (human inputs) due to a relatively small particle size (compared to sediments) [16,18]. On the other hand, as a vital pre-sink of PTEs in aquatic environment [14,17], High PTEs’ content SPM in the Jiulongjiang River is bound to intensify the PTE accumulation in fluvial bed sediment via the deposition process. Moreover, damming activities (hydropower reservoir) will further accelerate this process (deposition) by changing the hydrodynamic condition [25,26,27], which is adverse to the river eco-environment. Therefore, the PTEs’ geochemistry regarding fluvial SPM is a good indicator of the river eco-environment.

## 4. Conclusions

In conclusion, the PTEs’ investigation of suspended sediments in a coastal river (the Jiulongjiang River) was performed. The PTEs’ concentrations, contamination status, and risk were assessed, and the potential sources of PTEs and their behavior between different phases were also identified. The main findings revealed that the PTEs’ contents were in the order Mn > Zn > V > Pb > Cr > Ni > Cu > Cd > Co. For most PTEs, the EF, I_geo_, and PLI assessment pointed to no enrichment/unpolluted level, Pb and Zn exhibited minor enrichment/moderately polluted level, and Cd was positioned in the extremely severe enrichment/polluted level. Based on the TRI calculation, Cd and Zn were the two main toxic factors of suspended sediments, which contributed 57 ± 18% and 14 ± 7% of the total TRI. Principal component analysis provided some evidence that human input may be the major source of Cd, Zn, Pb, whereas Co. Cu, Cr, V, and Ni are mainly from natural processes (e.g., rock weathering), and Mn is primarily from the contribution of both natural and anthropogenic inputs. The PTEs’ behavior between phases indicated that SPM geochemistry can be a potential indicator of basin-scale environmental variations, and further study on PTEs of the suspended sediments would be valuable.

## Figures and Tables

**Table 1:** The particulate PTEs’ contents in JRB and world rivers (mg kg^−^^1^).

River	Parameter	V	Cr	Mn	Co	Ni	Cu	Zn	Cd	Pb
JRB-Entire Basin	Min	47.0	14.9	577.6	4.5	9.1	6.0	77.0	7.5	10.3
	Max	179.2	271.4	3041.7	23.5	91.0	98.5	1111.2	30.8	125.0
	Mean	95.3	60.3	1245.5	11.0	33.0	22.0	338.1	11.2	70.1
	Median	96.2	58.0	1127.1	10.3	31.3	19.3	309.3	10.8	70.0
JRB-Beixi River	Mean	104.1	62.8	1221.6	13.0	39.5	18.8	393.0	12.3	75.3
	Median	106.6	62.2	1085.2	13.9	40.1	18.2	391.7	11.7	76.2
JRB-Xixi River	Mean	76.8	40.3	1122.8	8.4	18.9	25.6	279.9	9.9	74.8
	Median	79.3	37.1	1029.3	8.1	18.5	21.7	258.0	9.8	69.8
JRB-Nanxi River	Mean	86.5	89.3	1671.9	8.4	34.5	33.9	228.6	10.0	48.3
	Median	60.0	35.4	1534.1	6.8	19.0	13.5	202.9	8.3	48.0
JRB-Estuary	Mean	100.1	65.1	1249.0	10.2	32.7	19.8	319.3	10.5	60.1
	Median	98.1	65.6	1311.5	10.5	31.8	20.4	307.3	10.5	61.3
World River	Mean	129	130	1679	22.5	74.5	75.9	208	1.6	61.1
UCC		97	92	774.5	17.3	47	28	67	0.09	17
TEL		—	43.4	—	—	22.7	31.6	121	0.99	35.8
PEL		—	111	—	—	48.6	149	459	4.98	128

Note: PTEs’ concentrations of World River are derived from [14]; UCC = upper continental crust [44]; TEL = threshold effect level, PEL = probable effect level [41]; —, data unavailable.

**Table 2:** The PTEs concentrations of SPM between JRB and global rivers (mg kg^−^^1^).

Rivers	V	Cr	Mn	Co	Ni	Cu	Zn	Cd	Pb
Jiulongjiang River	96.2	58.0	1127.1	10.3	31.3	19.3	309	10.8	70.0
Zhujiang River	150.5	147.7	1103.6	—	41.6	36.3	139	3.5	38.6
Mun River	109.1	100.1	4616.7	—	51.0	27.6	224	10.7	14.3
Asia (China) river	135.0	117.0	970.0	21.0	68.0	53.0	145	—	64.0
Asia (Russia) river	128.0	260.0	5767.0	30.0	123.0	145.0	300	—	35.0
South American river	131.0	79.0	700.0	16.0	46.0	59.0	184	—	76.0
North American river	188.0	115.0	1430.0	15.0	50.0	34.0	137	—	22.0
Africa river	116.0	130.0	1478.0	23.0	78.0	53.0	130	—	46.0
Europe river	85.0	164.0	1884.0	16.0	66.0	172.0	346	—	71.0
World river	129.0	130.0	1679.0	22.5	74.5	75.9	208	1.6	61.1

Note: Data of PTEs of other rivers are from [14,22,43]; —, data is not available.

**Table 3:** The CF and PLI evaluation results of PTEs of the SPM in Jiulongjiang River.

	Site	CF									PLI
		V	Cr	Mn	Co	Ni	Cu	Zn	Cd	Pb	
Beixi River	1	0.9	0.6	3.8	0.6	0.7	0.6	4.2	154.3	6.6	2.3
	2	0.8	0.3	1.1	0.3	0.3	0.3	2.7	103.2	3.5	1.2
	3	0.9	0.4	1.2	0.5	0.4	0.5	3.4	109.0	4.0	1.6
	4	1.2	1.2	1.5	1.0	1.6	1.3	16.6	342.0	5.6	3.7
	5	0.9	0.6	1.3	0.8	0.8	0.6	5.9	136.2	4.4	2.2
	6	1.5	1.3	1.0	0.5	1.3	0.7	4.7	125.7	5.4	2.4
	7	1.2	0.8	2.2	1.1	1.2	0.8	8.4	122.7	4.0	2.7
	8	1.2	0.9	2.1	1.0	1.2	0.8	7.7	126.6	4.3	2.8
	9	1.1	0.8	1.2	1.4	1.3	0.8	8.0	139.6	4.4	2.7
	10	1.1	0.7	1.4	0.9	0.9	0.7	6.5	133.4	4.6	2.4
	11	1.2	0.8	2.1	0.5	0.7	0.6	6.4	138.9	3.7	2.2
	12	1.1	0.7	1.3	0.9	0.9	0.7	6.2	130.0	5.4	2.4
	13	0.7	0.2	1.2	0.3	0.2	0.2	2.1	92.7	2.9	1.0
	14	1.4	0.7	1.0	0.7	0.8	0.7	6.1	149.0	5.2	2.3
	15	1.0	0.6	1.7	1.0	0.9	0.6	6.0	121.9	5.0	2.3
	16	0.8	0.4	1.4	0.4	0.4	0.4	2.2	103.0	2.0	1.3
	17	1.1	0.7	1.6	1.0	0.9	0.6	5.8	130.4	4.8	2.4
	18	1.2	0.7	1.4	0.8	0.9	0.7	5.8	133.4	4.8	2.3
	19	1.1	0.6	1.6	0.8	0.8	0.7	5.7	129.7	4.5	2.3
	20	1.1	1.0	1.3	0.7	1.0	0.6	5.7	127.6	4.5	2.3
	21	1.0	0.4	1.9	0.7	0.4	1.2	3.1	129.1	3.3	1.9
Xixi River	24	0.8	0.5	1.0	0.3	0.5	0.6	3.3	103.9	3.8	1.5
	25	0.8	0.3	1.0	0.3	0.3	0.6	3.3	99.8	3.5	1.4
	26	0.8	0.4	1.3	0.4	0.4	0.6	3.2	105.6	3.8	1.6
	27	0.9	0.4	1.6	0.5	0.4	0.7	3.7	115.6	4.2	1.7
	28	0.7	0.5	2.1	0.8	0.5	1.5	7.3	126.2	7.4	2.4
	29	0.8	0.4	1.6	0.6	0.4	0.8	4.5	117.7	4.5	1.8
	30	0.6	0.4	2.3	0.5	0.3	0.7	3.2	98.4	4.0	1.6
	31	0.8	0.4	1.2	0.5	0.4	0.9	4.0	117.1	4.8	1.8
	32	0.8	0.6	1.3	0.5	0.4	1.6	4.6	113.3	4.3	2.0
	33	0.7	0.5	1.0	0.5	0.5	1.1	4.5	102.9	3.8	1.8
Nanxi River	34	0.7	0.6	0.7	0.4	0.6	0.4	1.1	83.8	0.6	1.1
	35	0.5	0.2	1.8	0.3	0.2	0.5	3.5	95.7	3.0	1.2
	36	0.5	0.2	3.9	0.4	0.2	0.4	2.6	89.7	2.7	1.3
	37	1.8	3.0	2.2	0.9	1.9	3.5	6.4	176.6	5.1	4.2
Estuary	22	1.1	0.7	1.4	0.6	0.7	0.8	5.4	124.6	4.3	2.2
	23	1.1	0.6	1.7	0.6	0.8	0.7	5.8	126.8	3.8	2.2
	38	1.0	0.6	2.3	0.7	0.6	0.8	4.5	116.9	4.0	2.1
	39	1.1	0.7	1.7	0.6	0.7	0.7	5.1	118.2	3.3	2.1
	40	1.0	0.8	0.9	0.5	0.7	0.5	3.4	98.6	2.2	1.6
	41	1.0	0.7	1.7	0.6	0.7	0.7	4.6	116.0	3.6	2.1
	42	1.0	0.7	1.7	0.6	0.6	0.7	4.5	113.8	3.6	2.0

**Table 4:** Fraction of particulate PTEs’ transportation in the Jiulongjiang River.

Parameters	Unit	V	Cr	Mn	Co	Ni
Suspended phase	mg kg^−1^	96.2	58.0	1127.1	10.3	31.3
Dissolved phase	μg L^−1^	0.29	0.17	1.42	0.03	0.38
Fraction of particulate		82.8%	83.5%	92.1%	81.6%	54.7%

## Data Availability

The data presented in this study are available upon request from the corresponding author.

## Supplementary Materials

The following supporting information can be downloaded at: https://www.mdpi.com/article/10.3390/ijerph19074293/s1, Table S1: Contamination and toxic risk categories based on enrichment factor (EF), geo-accumulation index (I_geo_) and toxic risk index (TRI).
