# Supplementary material for: Suspended Sediments Quality Assessment in a Coastal River: Identification of Potentially Toxic Elements
Source: Int J Environ Res Public Health. 2022 Apr 3;19(7):4293. doi: 10.3390/ijerph19074293 (PMC8999047; doi:10.3390/ijerph19074293)
Supplement: Supplementary file 1 [file ijerph-19-04293-s001.zip › ijerph-1648285-supplementary.pdf]

Supplementary Materials

1. Supplementary tables

Table S1. Contamination and toxic risk categories based on enrichment factor (EF), geo-accumulation index (I_{geo}) and toxic risk index (TRI).

EF	Enrichment Level	I_{geo}	Pollution Intensity	TRI	Toxic Risk
<1	no enrichment	<0	unpolluted	<5	no toxic risk
1–3	minor enrichment	0–1	lightly polluted	5–10	low toxic risk
3–5	moderate enrichment	1–2	moderately polluted	10–15	moderate toxic risk
5–10	moderately severe enrichment	2–3	moderately to heavily polluted	15–20	considerable toxic risk
10–25	severe enrichment	3–4	heavily polluted	>20	very high toxic risk
25–50	very severe enrichment	4–5	heavily to extremely polluted		
>50	extremely severe enrichment	>5	extremely polluted		
